# Tumor immunomodulation by nanoparticle and focused ultrasound alters gut microbiome in a sexually dimorphic manner

**DOI:** 10.7150/thno.99664

**Published:** 2025-01-01

**Authors:** Akansha Singh, Sri Vidhya Chandrasekar, Vishnu Thayil Valappil, Joy Scaria, Ashish Ranjan

**Affiliations:** 1 Department of Radiation Oncology, UT Southwestern Medical Center, Dallas, TX 75390, USA.; 2 Department of Veterinary Pathobiology, Oklahoma State University, Stillwater, OK 74078, USA.

**Keywords:** Gut Microbiome, Immunomodulation, Nanoparticle, Focused Ultrasound, Sexual dimorphism

## Abstract

**Background:** Local immunomodulation with nanoparticles (NPs) and focused ultrasound (FUS) is recognized for triggering anti-tumor immunity. However, the impact of these tumor immunomodulations on sex-specific microbiome diversity at distant sites and their correlation with therapeutic effectiveness remains unknown. Here, we conducted local intratumoral therapy using immunogenic cell death-enhancing Calreticulin-Nanoparticles (CRT-NPs) and FUS in male and female mice. We identified immune-related microbiome populations, aiming to translate our findings into clinical applications.

**Methods:** CRT-NPs were synthesized by loading CRT-delivering plasmids into cationic liposomes. Local tumor therapy was performed using CRT-NP and FUS-based histotripsy (HT) on poorly immunogenic Mouse Oral Squamous Cell Carcinoma (MOC2) in the flank regions of male and female mice. Fecal samples were collected and analyzed before and three weeks post-treatment. The microbiome features were then correlated with immune cell dynamics within tumors and systemic cytokine responses to identify prognostic biomarkers in both male and female subjects.

**Results:** Intratumorally administered CRT-NP induced tumor remission and immune cell activation in both male and female mice, whereas HT was ineffective in males and showed efficacy only in females. *Turicibacter* and *Peptococcus* inversely correlated with tumor growth, while *Enterorhabdus*, *Subdologranulum*, *Desulfovibrio*, and *Aldercreutzia-Asaccharobacter* showed direct correlations with tumor growth. HT induced higher levels of *Turicibacter* in MOC2-bearing females, while males displayed increased *Enterorhabdus* and *Streptococcus* populations. Independent of sex, treatments promoting CD4+ T helper cells, functional CD8+ T cells, and total macrophage infiltration correlated with higher levels of *Gastrophilales*, *Romboutsia*, *Turicibacter*, and *Peptococcus*. Alternatively, *Enterorhabdus*, *Desulfovibrio*, *Streptococcus*, and *Staphylococcus* corresponded to poor treatment outcomes in both sexes.

**Conclusion:** An enhanced abundance of *Enterorhabdus*, *Desulfovibrio*, *Streptococcus*, and *Staphylococcus* in response to immunomodulatory therapies could serve as predictive biomarkers in a sex-independent manner. These findings could also be potentially extended to the realm of personalized interventions through fecal transplantations to reverse immunosuppressive phenotypes in males and improve patient outcomes.

## Introduction

In recent years, immunotherapy utilizing tumor immunomodulatory nanoparticles (NPs) has garnered significant attention due to their capacity to enhance anti-tumor immune responses 1. These NPs, encompassing lipid-based NPs (LNPs), polymeric NPs, gold NPs (AuNPs), and mesoporous silica NPs (MSNs), offer unique advantages such as precise targeting, controlled release, and versatility in payload delivery 2-5. Through diverse mechanisms, including the delivery of immunostimulatory agents, inhibition of immune checkpoints, and modulation of the tumor microenvironment, the NPs can bolster immune-mediated tumor eradication 6. Many NPs exploit immunogenic cell death (ICD), characterized by the release/expression of damage-associated molecular patterns (DAMPs) from dying tumor cells, thus activating anti-tumor immune responses. We recently developed a lipid-based CRT plasmid encapsulating NP (CRT-NP) that can directly upregulate CRT-based DAMP in tumor cells 7. Data suggest that upon local intratumor administration, CRT-NP improved immune-mediated tumor regression and survival outcomes in several preclinical models 8.

Like NPs, therapeutic devices, such as focused ultrasound (FUS), radiation, and lasers can treat solid tumors, while also modifying the tumor immune microenvironment. When FUS is applied to ablate tumor tissue, it induces localized cell death within the targeted region. This cell death results in the release of various tumor molecules/antigens from the dying tumor cells into the extracellular space 9. Specifically, focused ultrasound (FUS)-based histotripsy (HT) generates controlled microscale cavitation bubbles within the targeted tumor microenvironment (TME), resulting in mechanical disruption and fragmentation of the tumor cells for release of DAMPs, proteins, peptides, and other molecules that are recognized as foreign by the immune system 10. We have shown that FUS-based histotripsy represents a promising approach for not only achieving local tumor control but also for eliciting systemic anti-tumor immune responses, making it a valuable adjunct to cancer immunotherapy strategies 11.

Males and females exhibit distinctive physiological and biological differences, and sex based dysbiosis can significantly influence their anti-tumor immunity 12. Recent advancements in microbiome research have shown that gut microbiome is a sex dependent biological variable that could impact tumorigenesis 12. Sexual hormones such as estrogen and androgen could select for different gut microbiota resulting in substantial gut microbiota differences in male vs female 13. However, very little is known about the role of sexual dimorphism driven gut microbiome compositional differences on therapeutic efficacy of NPs and HT. Understanding the correlation between these sex-specific differences and microbiome plasticity could open new avenues for personalized immunotherapy, thereby advancing the frontiers of sex-personalized therapeutic strategies. In this study, we characterized the distant gut microbiome-local tumor axis in both male and female subjects using a poorly immunogenic MOC2 model following CRT-NP and FUS local treatments. Since both FUS and CRT-NP local treatments work via different mechanisms, we hypothesized that they would induce distinct sex-specific changes in the tumor-microbiome axis. Furthermore, we postulated that, irrespective of tumor types, our data mining would enable the identification of unique sex-independent microbial signatures that could potentially serve as future candidates for fecal transplantations to reverse immunosuppressive phenotypes in such scenarios.

Our data shows that immunomodulatory therapies based on CRT-NPs and HT indeed elicit distinct therapeutic outcomes in male and female mice, which are correlated with their respective microbiome signatures. Notably, our identification of the presence of sex-independent bacterial signatures can personalize such therapies and enhance outcomes in patients.

## Materials and Methods

### Materials

The lipids DOTAP (1,2-dioleoyl-3-trimethylammonium-propane) (cat#890890) and cholesterol (cat#700000) were purchased from Avanti Polar Lipids, Inc, Alabaster, AL. Nanoplasmid containing CRT gene (NTC 9385R-Calreticulin) controlled by the CMV promoter was obtained from Nature Technology Company, Lincoln. Chloroform (cat#C2432) was purchased from Sigma-Aldrich, St. Louis, MO, USA. HEPES, sterile 1M buffer (cat#J848), and Triton X-100 (cat#0694) were purchased from VWR life science, Radnor, PA, USA. Agarose Precast Gel (3% TBE EtBr Wide Mini Ready - 20-well) (cat#1613030) was purchased from Bio-Rad, Hercules, CA, USA. DMEM (cat#11965092), Fetal bovine serum, FBS (cat#10082147), Penicillin-Streptomycin (cat#15140122), PBS (cat#10010023), Collagenase IV (cat#17104019), were purchased from ThermoFisher/Gibco, Waltham, MA, USA. The following Fluorochrome-conjugated monoclonal antibodies (mAbs) for flow cytometry were purchased from BioLegend, San Diego, CA, USA: APC-Cy7 anti-CD45 (cat#103115), PerCP anti-CD3 epsilon (cat#100325), PE-Cy5 anti-CD4 (cat#100410), PE-Cy7 anti-CD8a (cat#100721), PE anti-Granzyme B (cat#372208), BV510 anti-IFNγ (cat#505841), BV605 anti-PD-1 (cat#135219), BV510 anti-Ly-6G (cat#127633), PE anti-Ly-6C (cat#128007), BV650 anti-CD11b (cat#101239), APC anti-CD206 (141707), BV785 anti-MHC II (cat#107645), BV421 anti-CD11c (cat#117329), AF488 anti-CD86 (cat#105017), PE-Cy7 anti-PDL1 (cat#124314), AF488 anti-FOXP3 (cat#126406) and BV711 anti-NK1.1 (cat#108745). 10X RBC Lysis Buffer (cat# 00-4300-54) was purchased from Invitrogen, Waltham, MA, USA. Mouse OSCC (MOC2) cell line (cat#EWL002-FP) was purchased from Kerafast, Boston, MA, USA. Liberase™ TM Research Grade (cat#5401119001) was procured from Millipore sigma, St. Louis, MO, USA. Transcription factor buffer set (cat#562574) was purchased from BD Biosciences, San Jose, CA, USA.

### Synthesis and characterization of CRT-NP

Unilamellar cationic liposomes were formulated by a thin-film hydration method as we previously published 8. Briefly, DOTAP and cholesterol at a 1:1 molar ratio were dissolved in chloroform, and the solvent was removed using a Rotary Evaporator (Heidolph, Wood Dale, IL, USA). The resulting lipid film was desiccated overnight and then hydrated using 10 mM HEPES buffer (pH 7.4) at 55 °C and sonicated to homogeneity. To ensure stability and uniform size, the liposome formulation was subjected to extrusion five times using an extruder with two stacked Whatman polycarbonate nanopore filters (200 nm pore diameter; GE Healthcare, Chicago, IL, USA). Subsequently, the liposome preparations were gently mixed with pDNA (CRT) solutions (1:20) in a total volume of 100 µl of CRT-NP lipoplex solution, and the mixture was incubated for 30 min at room temperature. Before and after mixing with the pDNA, the size, polydispersity index (PDI), and surface charge were assessed using dynamic light scattering (DLS) and zeta potential measurements with a Brookhaven ZetaPALS instrument (Holtsville, NY, USA). Plasmid encapsulation was determined by gel retardation assay.

### *In vivo* mice study and CRT-NP/FUS treatments

All animal-related procedures were approved and carried out under the guidelines of the Oklahoma State University Animal Care and Use Committee. 7-8 weeks old male and female C57BL/6 mice were purchased from Charles River Laboratories. For tumor inoculation, MOC2 cells derived from female mice at 70-80% confluency were harvested, washed, and diluted with sterile cold PBS to generate a dose of 1.5 × 10^5^ cells in 100 μL per mouse, and transplanted subcutaneously (s.c) into the right flank of 36 mice (18 males and 18 females) mice using a 27-gauge needle (BD, Franklin Lakes, NJ, USA). In our preliminary studies, nanoparticles (NP) with an irrelevant plasmid did not demonstrate efficacy. Therefore, we included untreated and HT as comparative controls for this study. Once the tumors reached a size of 40-60 mm^3^ (Day 7), male and female mice (n=6 mice/group) were randomly assigned to the following treatment groups: 1) Control, 2) CRT-NP, and 3) Histotripsy (HT) 14-17. Mice received three intratumoral injections of CRT-NP (20 µg plasmid DNA per injection) over one week, while the HT group received two HT treatments, spaced three days apart 7, 10. HT treatments covering 10-20% of tumor in anesthetized mice were administered using an Alpinion system (Bothell, WA, USA) with FUS transducer featuring a 1.5 MHz central frequency with following parameters: 5 Hz PRF, 1% duty cycle, 600 W acoustic power, 20 s treatment time per focal point. Tumor growth was monitored and measured daily using serial caliper (General Tools Fraction™, New York, NY, USA) and the tumor volume was calculated based on the formula (Length X Width^2^)/2, where length was the largest dimension and width was the smallest dimension perpendicular to the length.

### Fecal sample collection, storage, and microbiome analysis

Mice were allowed a 2-week acclimatization period and provided with a standardized diet for the study duration. Fecal pellets (4-6 pellets per mouse, n=5 mice per group) for microbiome analysis 15, 17 were obtained from individual mice kept in clean containers before inoculation and on the day of sacrifice and were preserved at -80°C until further processing. Fecal pellets were processed and analyzed for microbiome at ZymoBIOMICS® Targeted Sequencing Service, (Zymo Research Corporation, Irvine, CA, USA). Briefly, DNA extraction was performed using ZymoBIOMICS®-96 MagBead DNA Kit (Zymo Research, Irvine, CA, USA). Bacterial 16S ribosomal RNA gene targeted sequencing was performed using the Quick-16S™ NGS Library Prep Kit (Zymo Research, Irvine, CA, USA) and V3-V4 region of the 16S rRNA gene were amplified using PCR and targeted library sequence was prepared. The final library was sequenced on Illumina® MiSeq™ with a v3 reagent kit (600 cycles). Microbiome data was analyzed primarily using MicrobiomeAnalyst 18. Briefly, BIOM files were imported to MicrobiomeAnalyst web tool with Qiime v.1.9.1 for community profiling and comparative analysis. Data were filtered to remove low count (<20%) and low variance (<10%), and then scaled and normalized at the total sum scale. Community composition comparisons and Alpha diversity metrics were calculated on filtered reads using “Microbiome R packages” 19. Beta diversity was performed using ordination-based method followed by calculating the Bray-Curtis Index. Differential abundance analysis (DAA) was performed using DESeq2 20 and edgeR 21 with default parameters. The resulting FDR-corrected p-values were used for further analysis. Differences in taxonomy abundance were determined using Linear Discriminant Analysis Effect size (LEfSe) 22 employing the Kruskal-Walli's rank sum test. Euclidean distance was used for community clustering with the Ward algorithm. Correlation heatmap analysis was used to describe the relationship between immune cells, cytokines, tumor volume and specific taxa at genus level. The correlation analysis was based on the R stats package (version.4.02), Hmisc package, and heatmap package. Networks were constructed using igraph r package (https://igraph.org) and visualized with the interactive platform Gephi (https://gephi.org/) and Chord plot was generated using circlize R package. Data visualization package ggplot2 (https://github.com/tidyverse/ggplot2) and Microbiome Analyst internal scripts were used for plotting figures.

### Immune cell analysis of harvested tumors

Mice were sacrificed on Day 20 post-inoculation, and blood and tumor tissues were collected. Tumors were excised, weighed, and processed on the same day for flow cytometry. Tumors were minced and digested with 200 U per mL Collagenase IV solution followed by filtration through a 70 μm cell strainer (Corning Inc., Corning, NY, USA) to obtain single cell suspensions. Cells were treated with RBS lysis buffer for 20 mins at room temperature and then stained with specific anti-mouse fluorochrome-conjugated antibody combinations for 60 mins on ice in the dark to distinguish immune cell populations. To stain for intracellular markers, IFNγ, Granzyme-B (GZMB), CD206 and FOXp3, cells were washed after surface marker staining, fixed and permeabilized with a transcription factor buffer set (BD Biosciences), and incubated with intracellular antibody for 60 mins in the dark on ice. Stained cells were run in an LSRII flow cytometer (BD Biosciences). Compensations were performed with single-stained cells. FlowJo software v.10.2 (Treestar Inc., Ashland, OR, USA) and NovoExpress software v1.6.2 (Agilent, Santa Clara, CA, USA) were used for data analysis. Gating for different immune cell population were done as follows: CD45+ (Tumor infiltrating leukocytes; TILs), CD45+ CD3+ (Total T-cells), CD45+ CD3+ CD4+ CD8- (TH, CD4+ T helper cells), CD45+ CD3+ CD8- CD4+ FOXP3+ (Treg, Regulatory T-cells), CD45+ CD3+ CD4- CD8+ (TC, CD8+ T-cells), CD45+ CD3+ CD4- CD8+ GZMB+/IFNγ+ (Effector cytotoxic T-cells), CD45+ CD3+ CD4- CD8+ PD1+ (Exhausted T cell), CD45+ CD3- NK 1.1+ (NK cells), CD45+ CD3- NK 1.1+ GZMB+/IFNγ+ (Cytotoxic NK cells), CD45+ CD3- NK 1.1+ PD1+ (Mature NK cells) CD45+ CD11b- CD11c+ (DC, Dendritic cells), CD45+ CD11b- CD11c+ MHC-II+ CD86+ (Activated DCs), CD45+ CD11c- CD11b+ (Total Macrophages), CD45+ CD11c- CD11b+ CD86+ MHC-II+ (Activated M1 Macrophages), CD45+ CD11c- CD11b+ 206+ (M2 Macrophages), CD45+ CD11b+ Ly6CHi Ly6G- (Monocytic-Myeloid derived suppressor cells, M-MDSC) & Ly6CLo Ly6G+ (Polymorphonuclear-MDSC, PMN-MDSC).

### Serum cytokine analysis

Murine cytokines, chemokines, and growth factors were determined using the Luminex™ 200 system (Luminex, Austin, TX, USA) by Eve Technologies Corp (Calgary, Alberta) using mice serum. Eve technologies utilize the Mouse Cytokine/Chemokine 32-Plex Discovery Assay® Array (MD32) (MilliporeSigma, Burlington, Massachusetts, USA). The 32 cytokines included Eotaxin, G-CSF, GM-CSF, IFNγ, IL-1α, IL-1β, IL-2, IL-3, IL-4, IL-5, IL-6, IL-7, IL-9, IL-10, IL-12p40, IL-12p70, IL-13, IL-15, IL-17A, IP-10, KC, LIF, LIX, MCP-1, M-CSF, MIG, MIP-1α, MIP-1β, MIP-2, RANTES, TNFα, and VEGF-A, with assay sensitivity ranging from 3.2 to 21.0 pg/mL. The analysis utilized 2X diluted 50 μL of serum sample per mouse, and the results were presented in picogram per mL concentration.

### Statistical analysis

Statistical analyses were performed using GraphPad Prism 10.0 software (GraphPad Software Inc, La Jolla, CA, USA). Data are presented as mean ± SEM unless otherwise indicated. All microbiome-related analyses were carried out in R version 2024.4.2 and Microbiome Analyst in-built tests. Analysis of differences between 2 normally distributed test groups was performed using an unpaired t-test assuming unequal variance. The differences between the treatments compared to the untreated control were analyzed by two-way ANOVA. For analysis of three or more groups, one-way ANOVA with Fisher test was performed. P values less than 0.05 were considered significant and represented as *p<0.05, **p<0.005, ***p<0.0005, ****p<0.0001. Pearson's correlation coefficient and Spearman's rank correlation coefficient were utilized in this study. The co-occurrence event to be a robust correlation if the spearman's correlation coefficient (ρ) was both >0.7 and statistically significant (*P*-value <0.05).

## Results

### Healthy C57BL6 male and female mice showed distinct gut microbiome signatures

**Figure 1:**
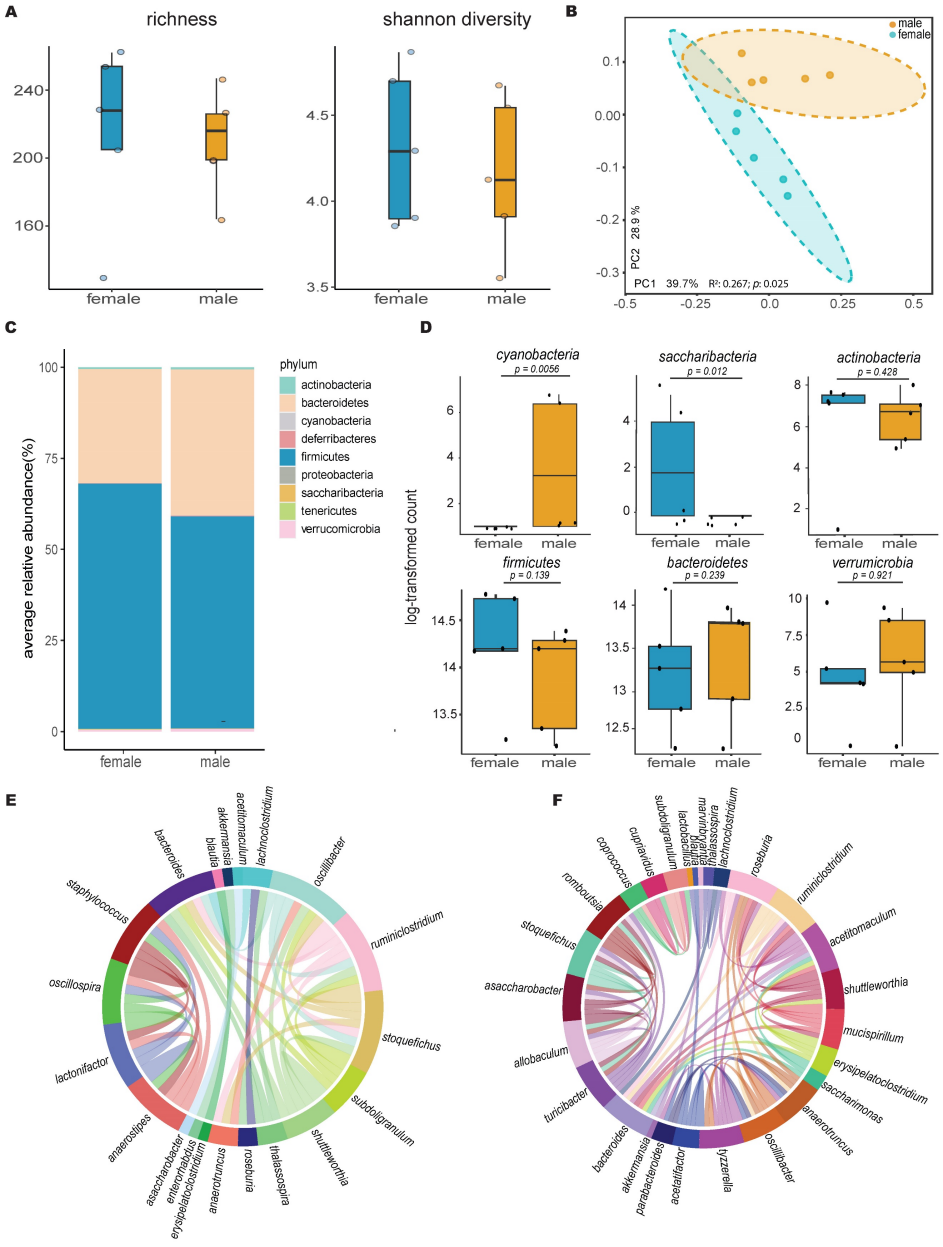
** Gut microbiota differs significantly between healthy female and male mice. A)** Alpha diversity calculations between healthy female and male mice, with each point representing an individual mouse in the group (n=5); **B)** Principal coordinate analysis of Bray-Curtis distances showing significant separation between the gender groups (PERMANOVA test values); **C)** Stacked bar plot illustrating the percent relative abundances of bacterial phyla between healthy female and male mice; **D)** Log-transformed counts of the most abundant and significant bacterial phyla between healthy female and male mice, with p-values determined using the Kruskal-Wallis test; **E & F**) Circos plots of Spearman correlations between genera in (E) male and (F) female mice. Each band represents the strength of the monotonic relationship between paired genera, with the Spearman correlation coefficient set at a minimum of 0.7 and the p value < 0.01.

Comparison of the gut microbiome between healthy male and female mice using 16S rRNA gene sequencing revealed higher bacterial diversity in the female group, as measured by two distinct metrics: Chao1 richness and Shannon diversity (Figure 1A). This was further supported by principal coordinates analysis of Bray-Curtis distances, which showed a significant separation between the gut microbiotas of male and female mice (n=5, p=0.025, R²=0.267, PERMANOVA; Figure 1B), with distinct compositional differences between the male and female groups (Figure 1C). Additional quantitative analysis of core microbiome abundance showed an abundance of OTUs in the *Verrucomicrobia* phylum distinctively in males (Figure S1).

Statistical comparison using the Kruskal-Wallis test of the most abundant phyla revealed that males had a significantly higher abundance of Cyanobacteria, while females had a higher abundance of Saccharibacteria (Figure 1D). Lefse analysis (LDA > 2 & FDR < 0.05) at the genus level identified two distinct populations, *Clostridium* and *Stoquefichus* (Figure S2), with healthy females showing a higher abundance of *Clostridium* (p=0.034) and males having a higher abundance of *Stoquefichus* (p=0.007).

Further analysis using Chord diagrams depicting genus-level Spearman correlation networks (p < 0.05) within male (Figure 1E) and female (Figure 1F) mice revealed distinct microbiome interaction patterns. In males, notable correlations were observed between several genera, with strong interactions among *Oscillibacter*, *Ruminiclostridium*, *Stoquefichus*, and *Bacteroides*, indicating a tightly interconnected network. In contrast, the female microbiome network showed a different set of correlations, particularly between *Roseburia*, *Ruminiclostridium*, *Bacteroides*, and *Oscillibacter*. Unique genera such as *Staphylococcus*, *Lactonifactor*, *Anaerostipes*, and *Enterorhabdus* were more prevalent in males, while *Turicibacter*, *Tyzzerella*, *Parabacteroides*, *Saccharimonas*, *Mucispirillum*, *Romboutsia*, and *Marvinbryantia* were predominant in females. Comparing the two networks, clear differences emerged in the central genera within the correlation structures. While some genera, such as *Ruminiclostridium* and *Bacteroides*, appeared in both networks, their interaction partners differed, reflecting gender-specific microbial community dynamics.

### Male mice displayed significantly faster MOC2 tumor growth compared to females with alterations in gut microbiome profiles

**Figure 2:**
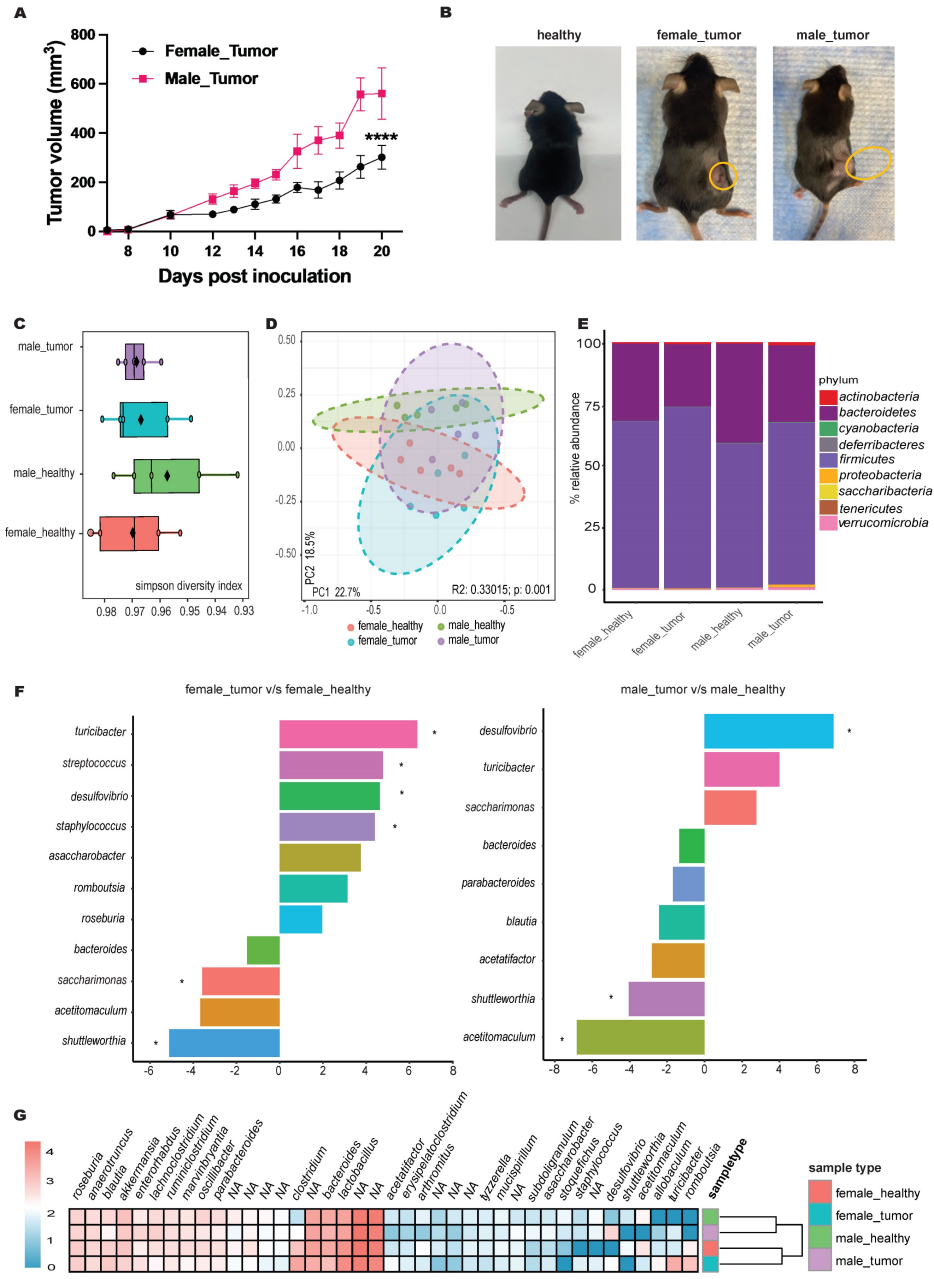
** MOC2 tumor growth pattern showed sex-based difference and shift in microbial abundance. A)** Line graphs depicting differential MOC2 tumor growth rates in male and female mice (n=6, ****p<0.0001, Two-way ANOVA with Tukey's multiple comparison); **B)** Representative images of healthy and tumor bearing female and male mice. **C)** Microbiome alpha diversity calculated using Simpson index (n=5); **D)** Principal coordinate analysis of Bray-Curtis distances shows significant separation between groups (PERMANOVA test values). Each point represents an individual's gut microbiota based on 16S sequencing; **E)** Bar plot of the relative genus-level abundance in healthy and tumor-bearing male and female mice; **F)** Differential abundance analysis of the microbiome between healthy and tumor-bearing male and female mice using edgeR (species with p < 0.05 shown, * significance at FDR < 0.05; tumor-bearing mice as baseline); **G) C**lustered heatmap of the top 40 genera for four groups of mice, with female and male (healthy and tumor-bearing) forming distinct clusters.

Male mice exhibited approximately 2-fold faster MOC2 tumor growth rates 10 days post-inoculation compared to females (Figure 2A-B). These changes were associated with shifts in microbiome alpha diversity compared to healthy mice (Figure 2C). For instance, the Simpson diversity index revealed lower microbial diversity in tumor-bearing mice, with principal component analysis (PCA) showing distinct clustering of microbial communities between healthy and tumor-bearing mice (p=0.001; Figure 2D).

Qualitative abundance analysis at the phylum level (Figure 2E) in both male and female tumor-bearing mice showed an increased Firmicutes-to-Bacteroidetes ratio, with males uniquely exhibiting elevated levels of Proteobacteria. EdgeR statistical comparison revealed that tumor inoculation caused more significant changes in genera abundance in females than in males (Figure 2F). Specifically, females showed an increase in *Turicibacter*, *Streptococcus*, and *Staphylococcus*, along with a decrease in *Saccharimonas*. In contrast, males exhibited a significant decrease in *Acetitomaculum*. Although not statistically significant, the abundance of *Saccharimonas* increased in males with tumors, opposite to the trend seen in females. Sex-independent changes included an increase in *Desulfovibrio* and a decrease in *Shuttleworthia*. To visualize the broader changes in tumor-bearing male and female mice compared to healthy controls, phenotype clustering using DESeq2 grouped the top 40 genera into male- and female-specific clusters (Figure 2G).

### In-situ immunomodulation with CRT-NP and HT induced distinct therapeutic responses between male and female mice

**Figure 3:**
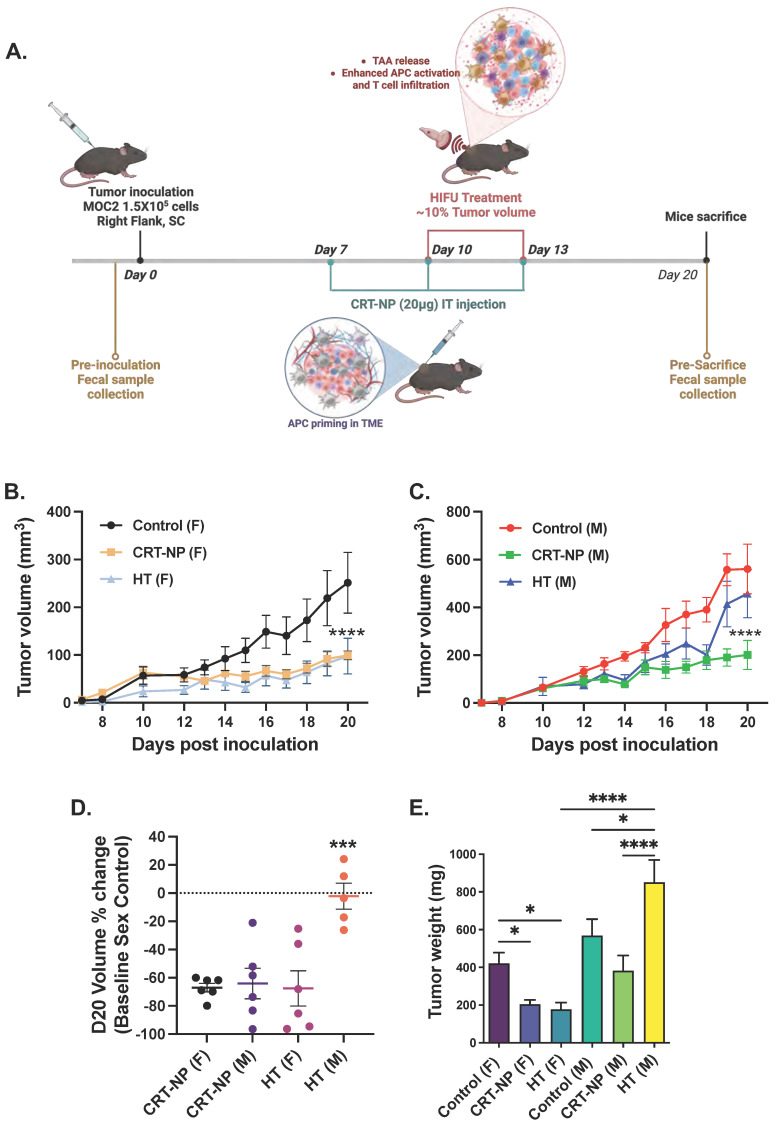
** CRT-NP & HT immunotherapies showed superior therapeutic response in female mice compared to male. A)** C57BL6 mice bearing unilateral flank tumors were treated seven days post-inoculation (n=6). Three intratumoral injections of CRT-NP at 20 µg CRT plasmid was performed. The HT group received two treatments covering 10-20% of the total tumor volume. Fecal samples were collected from all mice before inoculation and at the time of mice sacrifice (20 days post inoculation). **B&C)**, Line graphs representing MOC2 tumor growth post CRT-NP & HT treatments in female (B) and male (C). **D)** Median tumor regression percentage with CRT-NP & HT treatments in female and male mice compared to untreated control at the day of mice sacrifice (D20 post-inoculation). **E)** Tumors weights at the completion of study endpoint. Two-way ANOVA test was used to analyze tumor growth in line graph and one-way ANOVA was used for bar graphs analysis, * p<0.05, *** p<0.0005, **** p<0.0001.

Female mice bearing MOC2 tumors exhibited a significant reduction (~70%) in tumor growth when treated with both CRT-NP and HT therapies (Figure 3B-D). In contrast, among male mice, only CRT-NP displayed substantial therapeutic effectiveness, leading to a notable 60% decrease in both tumor growth and weights, mirroring the response observed in female mice. In contrast, HT treatment was ineffective in male mice (Figure 3C-E).

### Distinct sets of immune cells controlled the therapeutic outcomes of CRT-NP and HT therapies

**Figure 4:**
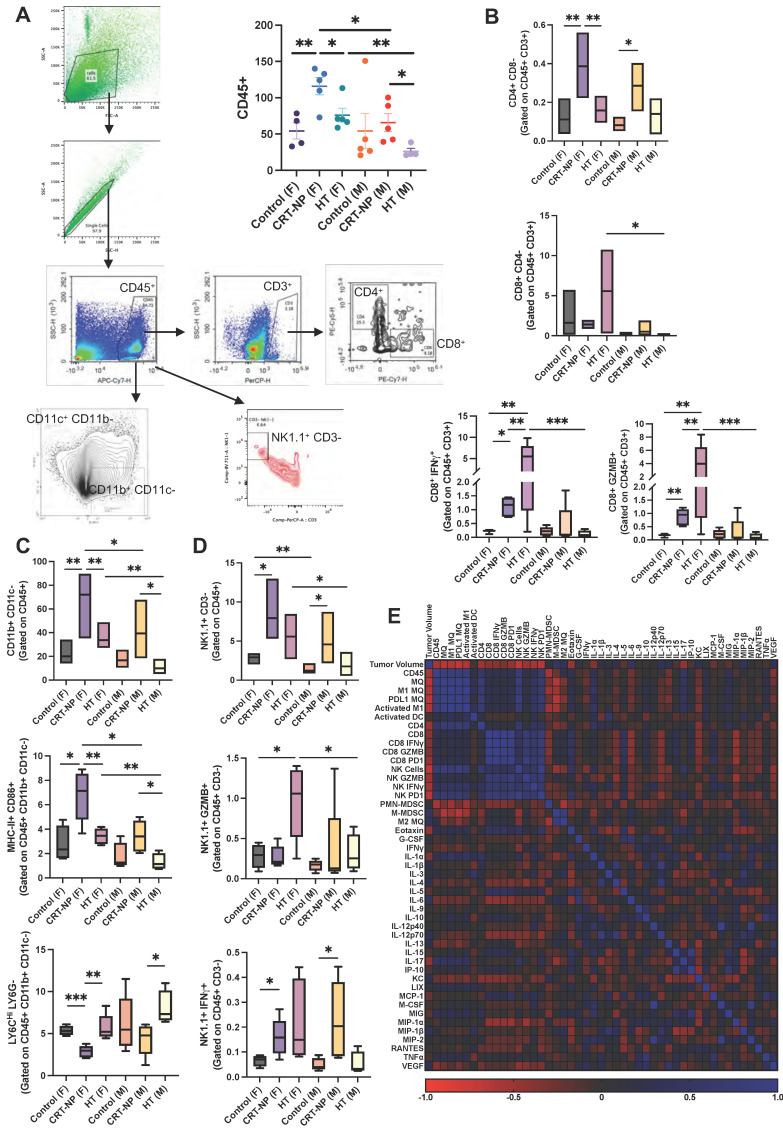
** CRT-NP & HT therapies efficacy is driven by distinct set of immune cells. A)** Flow cytometry analysis and gating strategy utilized to estimate distinct CD45+ immune cell number infiltrating MOC2 TME. **B)** Bar graph representing cell counts of CD8- CD4+ T helper cells, CD8+ CD4- cytotoxic T cells, and IFNγ and GranzymeB expressing CD8+ cytotoxic T cells, in TME of untreated, CRT-NP & HT treated tumors. **C)** Bar graph representing cell counts of CD11b+ CD11c- macrophages, MHC-II+ CD86+ M1 macrophages, Ly6Chi LY6G- M-MDSCs, in TME of untreated, CRT-NP & HT treated tumors of male and female mice. **D)** Bar graph representing CD3- Natural Killer cell counts, NK1.1+ CD3- NK cells, NK1.1+ GranzymeB+ cytotoxic NK cells, NK1.1+ IFNγ+ activated NK cells, in TME of untreated, CRT-NP & HT treated tumors of male and female mice. **E)** Pearson R correlation plot representing interaction profile of various TME associated immune cells, serum cytokines and chemokines levels and tumor volume. Immune cell counts are normalized and represented as cell number per mg of tumor. Difference among treatment groups (n=6) were analyzed using One-way ANOVA with Fisher test, * p<0.05, ** p<0.005, *** p<0.0005. P values for Pearson R correlation analysis is provided in Table 1. MQ: Macrophages, Activated M1: MHC-II+ CD86+ macrophages, Activated DC: MHC-II+ CD86+ dendritic cells (CD11c+ CD11b-).

Flow cytometry analysis of immune cell populations in male and female mice showed CRT-NP treatment was associated with an increase in CD45+ hematopoietic cell populations, whereas HT-treated males exhibited a decrease in this population (Figure 4A). Notably, CRT-NP treatment enhanced CD4+ T helper cell populations (3.5-4 folds) in both genders and promoted the infiltration of total macrophages (3.5 folds in female & 2 folds in male) and M1 macrophages (~3.5 folds in female & 2.5 folds in male) with decline (~2 folds in female) in M-MDSC infiltration within the tumor microenvironment (TME; Figure 4B-C). Conversely, HT-treated females exhibited the highest proportion of activated CD8+ cytotoxic T cells (16 folds) and NK cells expressing IFNγ (3 folds) and GZMB (4 folds), a phenomenon not observed in males across treatment groups (Figure 4B-D).

### Correlation of immune cells with serum cytokines

Expression of IFNγ and GZMB on CD8 T cells showed significant correlation with Eotaxin, MIP1α, KC, IL-5, IL-6. Whereas GZMB expression on NK cells showed significant correlation with IL-4 and MIP1α (Figure 4E). Pearson r correlation of various immune cells, cytokine and chemokines revealed significant correlation between IFNγ and PD1 expression on NK cells with total and activated macrophage population along with total and cytotoxic CD8+ T cells which also correlated with GZMB expression on NK cells.

### Gut microbiome alters with specific immunomodulatory treatment in sex dependent and independent manner

**Figure 5:**
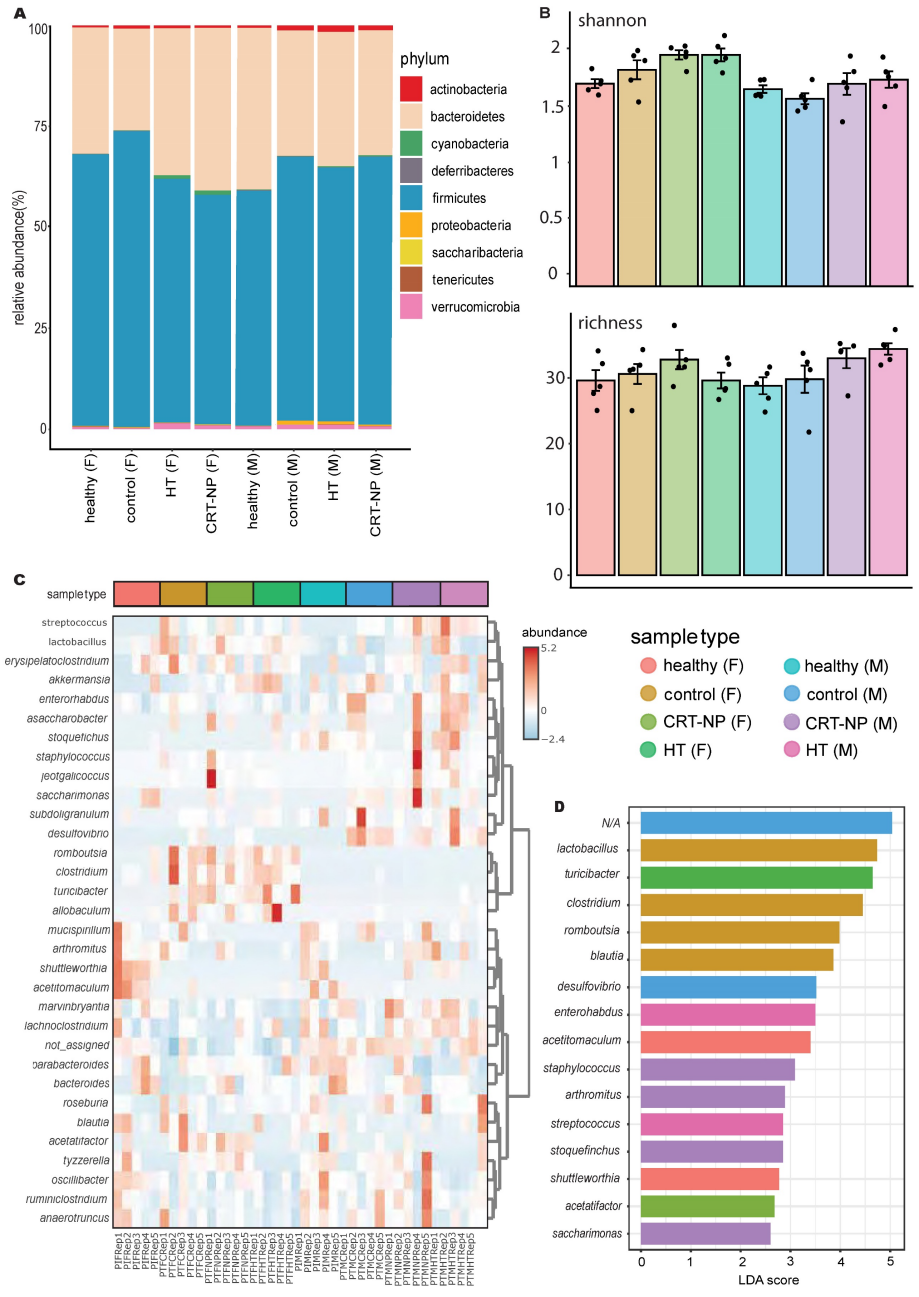
** Local immunomodulation alters gut microbiome and key genus abundance. A)** OTU clustering at the phylum level in healthy and tumor-bearing male and female mice following CRT-NP and HT treatments (n=5). **B)** Alpha diversity comparison between different groups calculated using Shannon and chao1 alpha diversity index. **C)** Hierarchical clustering and correlation analysis of OTUs at the genus level in different treatment groups. **D)** Bar graph representing top 15 populations identified with LEfSe analysis at the genus level across multiple treatments. The cutoff of LDA score in the LEfSe analysis is 2.0.

OTU clustering revealed a reduction in the Firmicutes to Bacteroidetes ratio and an increase in Cyanobacteria in females treated with CRT-NP and HT compared to untreated control and healthy mice. In contrast, males did not exhibit noticeable changes with treatment, except for a decrease in Proteobacteria populations in the CRT-NP treated group compared to other groups (Figure 5A). Both male and female mice exhibited increased alpha diversity with CRT-NP and HT treatments employing Shannon, Simpson and Chao1 matrix (Figure 5B). The hierarchical clustering and correlation analysis of OTUs at the genus level revealed distinct population patterns (Figure 5C). Further identification of distinct genera using LefSe analysis identified 32 genera with increasing LDA scores (>2), top 15 represented in Figure 5D. HT treatment in females induced higher accumulation of *Turicibacter* in females, while in males *Enterorhabdus* and *Streptococcus* were increased. With CRT-NP treatment, higher abundance of *Acetatifactor* was observed in females whereas *Staphylococcus*, *Arthromitus*, *Stoquefichus* and *Saccharimonas* were elevated.

### Specific microbial genera and immune features correlates with immunotherapeutic outcomes

**Figure 6:**
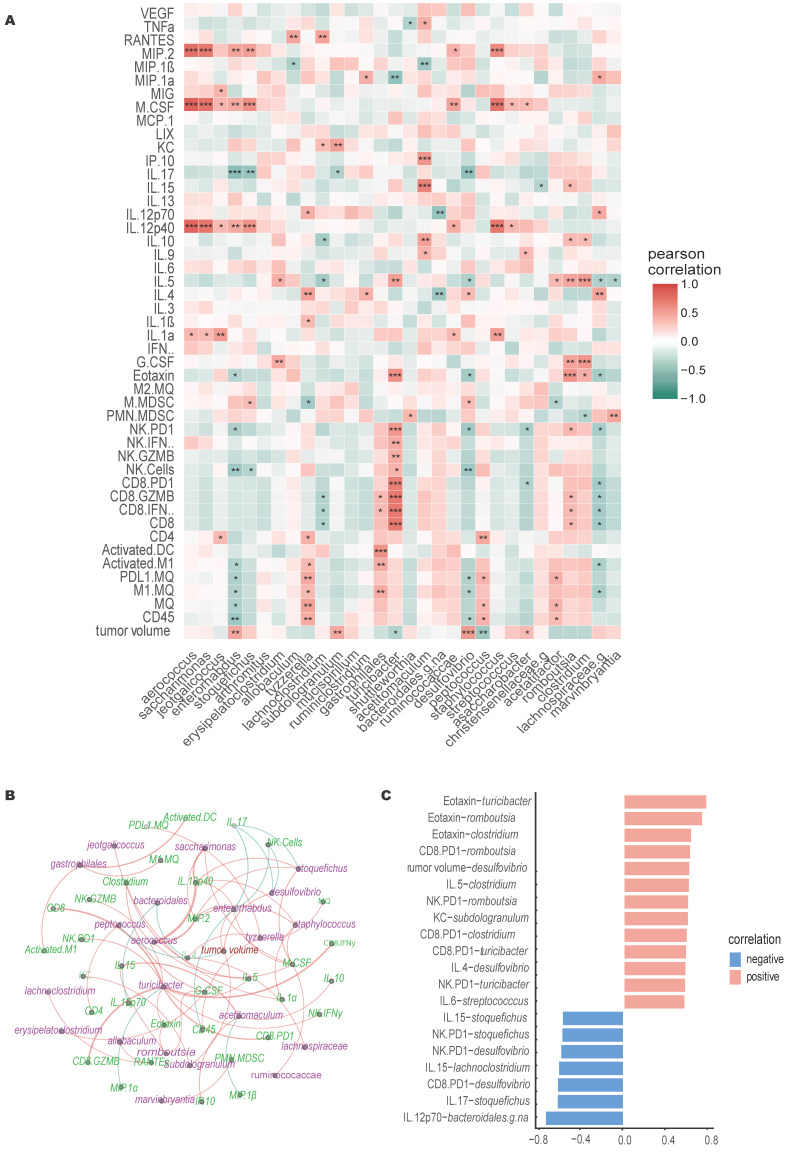
** Correlation of immune cells, cytokines, and gut microbiome. A)** Pearson correlation heatmap of genera with tumor burden, TME infiltrating immune cells, serum cytokines and chemokines levels, * significance at *P* < 0.05, ** significance at *P* < 0.01, and *** significance at *P* < 0.001, n=30**. B)** In the bacteria-immune correlation network for each treatment, only significant correlations were considered. Blue lines represent negative correlations, and orange lines represent positive correlations. The thickness of the lines indicates the strength of the correlation. **C)** Significant Pearson correlations for bacteria-immune pairs (p<0.001) are shown, with the x-axis representing the correlation coefficient and the y-axis representing the bacteria-immune pairs.

Multifactor LefSe comparison, identified a total of 32 genera that displayed LDA score >3.5. Pearson r correlation analysis conducted on 31 identified genera (excluding non-classified genus, NA) revealed both direct and inverse correlations with various immune cells, cytokines, chemokines, and tumor growth (Figure 6A-C). Notably, *Turicibacter* and *Peptococcus* exhibited an inverse relationship with tumor growth, while *Enterorhabdus*, *Subdologranulum*, *Desulfovibrio*, and *Adlercreutzia-Assacharobacter* were directly correlated with tumor growth.

Correlation with immune cell dynamics indicated that *Tyzzerella* and *Gastrophilales* showed positive correlation with M1 macrophage abundance in TME, whereas *Lachnospiraceae* and *Enterorhabdus* was inversely correlated. *Gastrophilales* also correlated positively with activated DC and cytotoxic CD8+ T cells (IFNγ+ & GZMB+) accumulation in TME. Similarly, *Turicibacter* showed positive correlation with higher CD8+ T cell and NK cell infiltration and activation in tumors. *Lachnoclostridium* and *Lachnospiraceae* showed inverse correlation with CD8 T cells cytotoxicity whereas *Romboutsia* showed positive correlation. Higher levels of *Jeotgalicoccus*, *Tyzzerella* and *Peptococcus* were associated with higher CD4+ T cells infiltration in tumors. *Enterorhabdus*, *Stoquefichus* and *Desulfovibrio* abundance was negatively correlated with NK cell tumor infiltration. Higher M-MDSC accumulation was associated with higher *Stoquefichus* and *Desulfovibrio*, and with lower *Tyzzerella* and *Acetatifactor*. Additionally, PMN-MDSCs accumulation were negatively correlated with *Clostridium* and positively with *Shuttleworthia* and *Marvinbryantia*.

*Acetitomaculum* and *Romboutsia* showed positive correlation with lymphocyte stimulating cytokines whereas *Aerococcus*, *Saccharimonas*, *Stoquefichus*, *Enterorhabdus*, *Ruminicocaccae*, *Staphylococcus*, *Streptococcus* positively correlated with inflammatory, pro-tumoral cytokines like IL-1, M-CSF, MIP-2, IL12p40 or IL-17, etc. Similar to tumor growth, *Desulfovibrio* and *Turicibacter* showed contrasting correlation with IL-5 and Eotaxin serum levels. *Desulfovibrio* also correlated with significantly with CD4+ T cells associated cytokines, positively with IL-4 levels but inversely with IL-17 levels. On the other hand, higher *Turicibacter* were associated with lower pro-tumoral MIP-1α levels.

## Discussion

Changes in alpha and beta microbiome diversity, are known to be strongly associated with altered anti-tumor immunity 23. While the influence of sexual hormones on immune responses and microbiome dynamics is recognized 24, 25, the extent to which microbiome signatures induced by specific treatments (such as CRT-NP and HT) consistently appear across genders, or operate independently, remains uncertain. This understanding is crucial for customizing personalized solid tumor immunotherapy regimens.

We first profiled the gut microbiomes of healthy, non-tumor-bearing male and female mice. At the phylum level, females exhibited a higher Firmicutes-to-Bacteroidetes ratio, consistent with previous studies 26. Additionally, we observed elevated levels of *Saccharibacteria* in females, which has been associated with higher sugar metabolism 27, 28 and protection from inflammatory damage induced by pathogens in mammalian hosts 29. In contrast, the gut microbiome of males showed a significantly higher presence of *Cyanobacteria*, whose derivatives and metabolites are currently being studied for their anti-tumor properties 30. However, it is worth noting that cyanobacterial blooms, particularly their toxins—such as microcystins, cylindrospermopsin, and lipopolysaccharides—are known to contribute to gastric cancers 31. Given the contradictory outcomes of HT in males, we hypothesize that its efficacy may be treatment-dependent. Among various phyla, *Firmicutes* emerged as a key regulator of sex-dependent disparities, as most genera showing differences between males and females belonged to this phylum. For example, *Staphylococcus*, *Lactonifactor*, and *Anaerostipes* were more abundant in males, while *Turicibacter*, *Tyzzerella*, *Romboutsia*, and *Marvinbryantia* were more prevalent in females.

The MOC2-bearing male mice demonstrated rapid tumor progression consistent with poorly immunogenic profiles, as observed in other models 32, 33. Since the MOC2 cell lines used in this study were derived from female mice, it was surprising to find that the tumors grew faster in males, as they should ideally have grown slower due to tumor antigen mismatch between sexes. Prior research indicates that oropharyngeal squamous carcinoma with higher estrogen receptor α (ERα) expressions is associated with improved survival among those receiving chemoradiation 34. While the ERα expression profile of MOC2 is not known, the improved outcome in females vs. males suggests potential hormonal signaling influencing growth patterns. We also examined the tumors of untreated male and female mice and observed moderately higher CD8 T cell and NK cell infiltration in female tumors. These observations led us to hypothesize that microbiome-related factors between sexes could be at-play, which we investigated in this study.

Our data suggested that NK cell infiltration correlated with four bacterial genera, among which *Desulfovibrio* (DSV) was persistently higher in males. DSV has been shown to be in higher abundance in gastric, colon, rectal, and breast cancer patients 35, inducing colonic damage and pre-metastatic niche in mice liver by increasing IL-1β, TNFα, CXCL12 and various matrix metalloproteases 36. We found that DSV, similarly, correlated positively with faster tumor growth, higher serum IL-4 and reduced Eotaxin and IL-17 levels (Figure 6). In addition to DSV, three other genera (*Adlercreutzia-Asaccharobacter*, *Subdoligranulum*, and *Enterorhabdus*) showed a positive correlation with tumor growth, while *Turicibacter* and *Peptococcus* exhibited a negative correlation. Previous studies have linked a decrease in *Peptococcus* abundance with tumor growth in esophageal squamous cell carcinoma 37, prostate cancer patients 38, and cachectic cancer patients 39. Chen *et al.* demonstrated that higher gut *Peptococcus* abundance decreases the risk of non-small cell lung cancer via CD4+ T cells 40. Similarly, we observed a positive correlation between *Peptococcus* abundance and CD4+ T cell infiltration in MOC2 tumors. Interestingly, *Peptococcus* also correlated with increased macrophage numbers in the TME and higher PD-L1 expression on macrophages, potentially limiting its use as a predictive marker for successful immunotherapy outcomes.

Finally, we assessed the microbiome dynamics in MOC2-bearing mice treated with CRT-NP and FUS in both sexes. The intratumoral route of CRT-NP administration was selected to explore how localized treatment of a distant tumor affects gut microbiome abundance. This approach contrasts with other studies that have focused on tumor cell and tumor microenvironment (TME) resident microbiome interactions 41-43, or on gut microbiome modulation through systemic treatments 44, 45. However, the impact of localized tumor treatment on the gut microbiome, particularly in distant tumors, remains underexplored. CRT-NP induced a similar decline in MOC2 tumor growth in both sexes. However, HT failed to control MOC2 tumor growth in males, while efficiently retarding tumor growth in females, mirroring the efficacy of CRT-NP therapy (Figure 3). Unlike caliper measurements of tumor volume, males showed higher tumor weight compared to untreated controls (Figure 3C-E), suggesting a faster inward spread of the tumor toward the peritoneal space by the day of sacrifice, which was not fully captured by caliper measurements. To elucidate the potential immune-mediated mechanisms underlying these outcomes, we examined the tumor immune microenvironment and systemic cytokine profiles. We observed an increase in CD4+ T cells and macrophages in both sexes following CRT-NP treatments, with levels higher in females. Tumor-infiltrating macrophages displayed polarization toward the M1 type, accompanied by a decrease in MDSC population. In contrast, with HT, TME immune cell profiling showed a higher engagement of cytotoxic NK cells and CD8 T cells in HT-treated female tumors compared to all other groups (Figure 4). Pearson r analysis showed strong correlation of cytotoxic T cells and NK cells with *Turicibacter* abundance (Figure 6C) which increased significantly in HT treated females. In contrast, genera like *Streptococcus* and *Enterorhabdus* were highly abundant in HT-treated male mice (non-responders) and showed a positive correlation with serum IL-1, M-CSF, MIP-2, IL12p40 or IL-17, pro-tumoral cytokines, and chemokines. *Streptococcus* and *Staphylococcus* species are well-known to promote the development of cancer in various tissues 46, 47. A retrospective analysis of the intestinal microbiome composition in 44 pancreatic cancer patients demonstrated a significant increase in *Streptococcus* content 48. Their higher abundance was also noted in non-responder NSCLC patients receiving anti-PD1 therapy 49. *Enterorhabdus* has been associated with ileocecal inflammation 50, and its gut levels negatively correlated with IFNγ serum levels 51. In addition to protumoral cytokines, we observed *Enterorhabdus* showed a negative correlation with intratumoral M1 macrophage activation and NK cell numbers (Figure 6). Additionally, a positive correlation of *Turicibacter* with cytotoxic T cells and NK cell populations in the tumor microenvironment as well as serum levels of Eotaxin and IL-5 indicate a treatment-specific change in the gut microbiome (Figure 6A-C). Recent research has suggested that *Turicibacter* plays a role in creating a favorable tumor microenvironment by enhancing the production of metabolites, such as short-chain fatty acids (SCFAs), which can improve the recruitment and activation of immune cells like cytotoxic T lymphocytes and natural killer cells 52, 53.

Our study has some limitations. We used a single tumor line in a single mouse strain. Panshak *et al.* previously demonstrated that females showed a higher response to the B16-F10/BL6 syngeneic mouse model melanoma than males 32. Thus, as evidenced by our correlative analysis and prior reports, our findings could be generalized based on sex with the added element of microbiome signatures, along with cytokine and immune cell features. Furthermore, the translational impact of our findings would be best demonstrated by microbial transplantation from female FMT into untreated males to show reduced tumor growth. The goal of this study was to highlight and identify sexually dimorphic signatures indicative of microbiome-correlated outcomes. Future studies performing microbial depletion experiments or fecal transplantations to validate these findings would provide more direct evidence of the causal relationship between these microbial species and treatment response. However, those studies were beyond the scope of this paper.

## Conclusion

To summarize, our study highlights the potential significance of sex-specific factors and microbiota composition in treatment response. Particularly, higher levels of *Enterorhabdus*, DSV, *Streptococcus* and *Staphylococcus* in fecal samples after NP and HT immunomodulatory treatment can serve as predictive biomarker for treatment response, independent of sex. The differential response observed in male mice with HT underscores the importance of considering sex-specific factors and associated microbiota composition in personalized therapies. Characterizing biomarkers before treatment initiation can aid in the selection and stratification of male and female patients who are expected to respond most favorably to treatments.

## Supplementary Material

Supplementary figures.

## Figures and Tables

**Table 1:** P value for Pearson R correlation between tumor burden, TME infiltrating immune cells and serum cytokines and chemokines level.

Variables	Correlation score	P-value	Variables	Correlation score	P-value	Variables	Correlation score	P-value
Volume-CD45	-0.52	0.00421634	PDL1 MQ-M-MDSC	-0.61	0.00055154	CD8 PD1-IL-6	-0.37	0.04659335
Volume-MQ	-0.62	0.00039106	PDL1 MQ-M2 MQ	-0.37	0.04993837	CD8 PD1-MCP-1	0.44	0.01763022
Volume-M1 MQ	-0.58	0.00106689	PDL1 MQ-IL-17	0.44	0.01849909	NK cells-NK GZMB	0.49	0.00683037
Volume-PDL1 MQ	-0.66	0.00011364	Activated M1-Activated DC	0.39	0.03919269	NK cells-NK IFNγ	0.53	0.00357604
Volume-Activated M1	-0.57	0.00144691	Activated M1-CD4	0.67	7.1165E-05	NK cells-NK PD1	0.91	1.7061E-11
Volume-CD4	-0.48	0.00920969	Activated M1-NK Cells	0.68	5.9748E-05	NK cells-M-MDSC	-0.41	0.02738338
Volume-CD8	-0.38	0.04219493	Activated M1-NK IFNγ	0.47	0.01141053	NK cells-Eotaxin	0.43	0.02103778
Volume-CD8 PD1	-0.42	0.0236728	Activated M1-NK PD1	0.56	0.00185548	NK cells-IL-17	0.48	0.00939359
Volume-NK Cells	-0.67	8.7192E-05	Activated M1-PMN-MDSC	-0.46	0.01318932	NK cells-VEGF	-0.4	0.03228272
Volume-NK GZMB	-0.47	0.01109233	Activated M1-M-MDSC	-0.49	0.00743	NK GZMB-NK IFNγ	0.64	0.0002028
Volume-NK IFNγ	-0.52	0.00386049	Activated M1-IL-1α	0.47	0.01147772	NK GZMB-NK PD1	0.69	3.6844E-05
Volume-NK PD1	-0.64	0.0002259	Activated M1-IL-17	0.39	0.03591859	NK GZMB-IL-4	-0.39	0.03697605
Volume-M-MDSC	0.47	0.01051002	Activated DC-IL-3	0.52	0.0050792	NK GZMB-MIP-1α	-0.48	0.01225285
Volume-M2 MQ	0.39	0.03869564	CD4-NK Cells	0.62	0.00043071	NK IFNγ-NK PD1	0.63	0.00032468
Volume-Eotaxin	-0.44	0.01887789	CD4-NK IFNγ	0.43	0.02159545	NK PD1-Eotaxin	0.5	0.00603451
Volume-IL-6	0.43	0.02113829	CD4-NK PD1	0.43	0.02095208	PMN-MDSC-M2 MQ	-0.52	0.00399718
Volume-IL-17	-0.41	0.02676211	CD4-IL-1α	0.43	0.02050272	M-MDSC-M2 MQ	0.8	2.3522E-07
Volume-MIP-1β	0.37	0.04801781	CD8-CD8 IFNγ	0.99	1.4308E-29	M-MDSC-IL-17	-0.43	0.0216771
CD45-MQ	0.84	2.1345E-08	CD8-CD8 GZMB	0.98	9.2678E-22	Eotaxin-IL-1α	0.4	0.03247099
CD45-M1 MQ	0.81	1.2673E-07	CD8-CD8 PD1	0.95	1.5493E-15	Eotaxin-IL-5	0.43	0.02068092
CD45-PDL1 MQ	0.79	3.6753E-07	CD8-NK Cells	0.44	0.0184868	Eotaxin-IL-15	0.44	0.0167475
CD45-Activated M1	0.8	2.1758E-07	CD8-NK GZMB	0.69	4.4474E-05	Eotaxin-IL-17	0.38	0.04066252
CD45-CD4	0.53	0.00352439	CD8-NK IFNγ	0.59	0.0008205	Eotaxin-MIP-1β	-0.38	0.04307274
CD45-NK Cells	0.68	6.0604E-05	CD8-NK PD1	0.72	1.0613E-05	G-CSF-IL-5	0.48	0.0096817
CD45-NK PD1	0.58	0.00098454	CD8-Eotaxin	0.42	0.02528201	G-CSF-IL-6	0.61	0.00055369
CD45-PMN-MDSC	-0.52	0.00450039	CD8-IL-5	0.41	0.02905943	G-CSF-IL-10	0.47	0.01051729
CD45-IL-17	0.48	0.00864797	CD8-IL-6	-0.39	0.03618232	IL-1α-IL-12p40	0.46	0.01725341
MQ-M1 MQ	0.93	8.0992E-13	CD8-MIP-1α	-0.43	0.02544701	IL-1α-IL-15	0.41	0.02979496
MQ-PDL1 MQ	0.98	5.3277E-22	CD8 IFNγ-CD8 GZMB	0.99	3.5215E-28	IL-1α-M-CSF	0.48	0.0093922
MQ-Activated M1	0.92	4.0689E-12	CD8 IFNγ-CD8 PD1	0.93	5.0268E-13	IL-1α-MIG	0.52	0.00438417
MQ-CD4	0.7	3.0944E-05	CD8 IFNγ-NK Cells	0.42	0.02283366	IL-1α-MIP-2	0.39	0.03521646
MQ-NK Cells	0.8	3.1608E-07	CD8 IFNγ-NK GZMB	0.67	9.5083E-05	IL-1β-IL-4	0.64	0.00022742
MQ-NK IFNγ	0.45	0.01397965	CD8 IFNγ-NK IFNγ	0.58	0.00102969	IL-1β-IL-10	0.4	0.03243689
MQ-NK PD1	0.63	0.00031533	CD8 IFNγ-NK PD1	0.71	1.6229E-05	IL-1β-IL-12p70	0.72	3.7999E-05
MQ-M-MDSC	-0.59	0.00074024	CD8 IFNγ-Eotaxin	0.43	0.0220556	IL-3-MIP-1β	0.57	0.00159166
MQ-IL-1α	0.37	0.04861054	CD8 IFNγ-IL-5	0.41	0.02891788	IL-4-IL-12p70	0.69	0.0001086
MQ-IL-17	0.43	0.0206379	CD8 IFNγ-IL-6	-0.38	0.0404886	IL-10-TNFα	0.63	0.00028375
M1 MQ-PDL1 MQ	0.9	4.9573E-11	CD8 IFNγ-MIP-1α	-0.43	0.02824918	IL-10-VEGF	0.44	0.01678669
M1 MQ-Activated M1	0.99	1.7604E-33	CD8 GZMB-CD8 PD1	0.9	7.0141E-11	IL-12p40-M-CSF	0.96	2.5473E-15
M1 MQ-CD4	0.69	4.7562E-05	CD8 GZMB-NK Cells	0.39	0.03736217	IL-12p40-MIP-2	0.86	1.2327E-08
M1 MQ-NK Cells	0.7	2.3297E-05	CD8 GZMB-NK GZMB	0.62	0.00041501	IL-12p70-MIP-1β	0.47	0.01655269
M1 MQ-NK IFNγ	0.48	0.00897125	CD8 GZMB-NK IFNγ	0.56	0.00182819	IL-13-KC	-0.41	0.02728385
M1 MQ-NK PD1	0.59	0.00086081	CD8 GZMB-NK PD1	0.68	5.293E-05	IL-13-VEGF	0.53	0.0031358
M1 MQ-PMN-MDSC	-0.46	0.01222074	CD8 GZMB-Eotaxin	0.44	0.01814214	IL-15-IP-10	0.81	1.3313E-07
M1 MQ-M-MDSC	-0.48	0.00841607	CD8 GZMB-IL-5	0.42	0.02538759	IL-15-MIG	0.44	0.01703965
M1 MQ-IL-1α	0.46	0.01251544	CD8 GZMB-IL-6	-0.38	0.04224085	IL-15-MIP-1β	-0.44	0.01629144
M1 MQ-IL-17	0.4	0.03272108	CD8 GZMB-MIP-1α	-0.41	0.03601448	IL-17-MIP-1β	-0.4	0.03257627
PDL1 MQ-Activated M1	0.88	2.5978E-10	CD8 PD1-NK Cells	0.45	0.01491434	IP-10-MIG	0.59	0.0008301
PDL1 MQ-CD4	0.69	3.4299E-05	CD8 PD1-NK GZMB	0.77	1.0194E-06	KC-MIP-1α	-0.39	0.04445848
PDL1 MQ-NK Cells	0.85	7.414E-09	CD8 PD1-NK IFNγ	0.63	0.00031228	MCP-1-TNFα	0.54	0.00276259
PDL1 MQ-NK IFNγ	0.47	0.01013809	CD8 PD1-NK PD1	0.72	1.3719E-05	M-CSF-MIP-2	0.87	1.0176E-09
PDL1 MQ-NK PD1	0.69	3.9799E-05	CD8 PD1-IL-5	0.38	0.0429076	TNFα-VEGF	0.42	0.0240414

**Table 2:** Multifactor LefSe analysis details comprising P values, FDR and LDA score.

	Pvalues	FDR	Female_Pre-Inoculation	Male_Pre-Inoculation	Female_CRT-NP_treated	Female_Control	Male_CRT-NP_treated	Male_ Control	Female_HT_treated	Male_HT_treated	LDA score
*Desulfovibrio*	5.74E-05	0.0018381	0	0	8923.2	13121	23937	65572	0	52876	4.52
*Clostridium*	0.00024135	0.0038616	144010	2235.3	496230	565880	17645	21349	520580	20501	5.45
*Romboutsia*	0.000450.00045025	0.0048026	24719	0	158840	192030	984.86	0	178310	0	4.98
*Shuttleworthia*	0.00096931	0.0066677	11787	5221.1	0	0	2607.7	0	0	2062.6	3.77
*Streptococcus*	0.0010418	0.0066677	0	0	5280.6	8284.8	11392	2751.9	3833.4	13979	3.84
* Turicibacter*	0.0022415	0.011955	4654.3	0	482360	445440	6643.5	8649.2	892120	55124	5.65
NA	0.0064335	0.02941	7217100	7732300	6081800	5922800	7172200	7935700	5763100	7419200	6.04
*Stoquefichus	0.0091161	0.032931	620.57	11988	0	0	14034	5291.2	3409.9	13328	3.85
*Acetitomaculum*	0.0092618	0.032931	50578	29897	0	5320	1679.9	0	3477.8	0	4.4
*Acetatifactor*	0.032508	0.10403	2206.8	6379.8	10709	10133	1481.7	1102.3	1425.1	2209	3.68
*Staphylococcus*	0.036809	0.10708	0	0	13984	6369.6	24300	4299.5	0	15509	4.08
*Saccharimonas	0.045966	0.14924	3567.4	0	1827.9	0	7857.8	3684.3	841.96	1785.9	3.59
Blautia	0.075145	0.17052	100250	88686	8505.8	151680	30877	22304	55417	93517	4.85
*Enterorhabdus*	0.07573	0.17052	33655	35822	38280	61203	70522	92715	32401	95952	4.5
Lactobacillus	0.079934	0.17052	1277100	780290	1692900	1869000	1415600	780770	1662800	1123500	5.74
*Arthromitus	0.091419	0.18284	15928	8667.3	6396.2	7877.8	16403	1317.7	1003.2	5054.9	3.89
*Marvinbryantia*	0.12256	0.21885	53581	55119	33252	30160	62123	61358	13895	61901	4.38
*Tyzzerella*	0.12841	0.21885	6650.5	7494.2	9120.3	4228.4	9373.7	5807.6	0	2565.3	3.67
*Mucispirillum*	0.12994	0.21885	11109	10519	0	8302.8	6782.8	7310.1	0	1727.7	3.74
Akkermansia	0.14236	0.22777	49596	73143	100480	26760	74688	120500	155060	129800	4.81
*Oscillibacter*	0.15956	0.23488	74674	74724	40051	41532	66715	48148	25458	27517	4.39
*Jeotgalicoccus*	0.16148	0.23488	0	0	41366	1698.6	24765	4616.9	5984.3	16919	4.32
*Bacteroides*	0.17131	0.23834	570990	617050	478070	246170	479380	400770	372240	378120	5.27
*Parabacteroides*	0.20288	0.25587	66808	76631	50495	40998	38219	37863	53454	33063	4.34
Erysipelatoclostridium	0.2051	0.25587	5843.2	2158	6741.9	8117.6	4450.7	1752.3	5401.9	9518.6	3.59
Adlercreutzia_Asaccharobacter	0.2079	0.25587	0	2739.4	10338	3593.1	9115.1	8772.8	4061.2	21234	4.03
Anaerotruncus	0.24641	0.28779	70138	66565	37435	71962	72789	81796	22867	47024	4.47
*Roseburia*	0.25182	0.28779	24690	77833	34194	92600	83066	77479	47523	115050	4.65
*Lachnoclostridium*	0.26355	0.29082	123250	164210	110920	96061	171100	132620	109940	183460	4.64
Allobaculum	0.3536	0.37717	4188.9	0	16492	28145	0	1865.5	43499	7153.8	4.34
*Ruminiclostridium*	0.38684	0.39932	50676	65313	23773	37873	77226	53185	18436	41748	4.47
Subdoligranulum	0.78416	0.78416	1610.4	5052.1	1238.7	2625	2115.9	10619	3477.6	8565.9	3.67
