# Supplementary material for: Tumor immunomodulation by nanoparticle and focused ultrasound alters gut microbiome in a sexually dimorphic manner
Source: Theranostics. 2025 Jan 1;15(1):216–32. doi: 10.7150/thno.99664 (PMC11667224; doi:10.7150/thno.99664)
Supplement: Supplementary file 1 — Supplementary figures. [file thnov15p0216s1.pdf]

S1

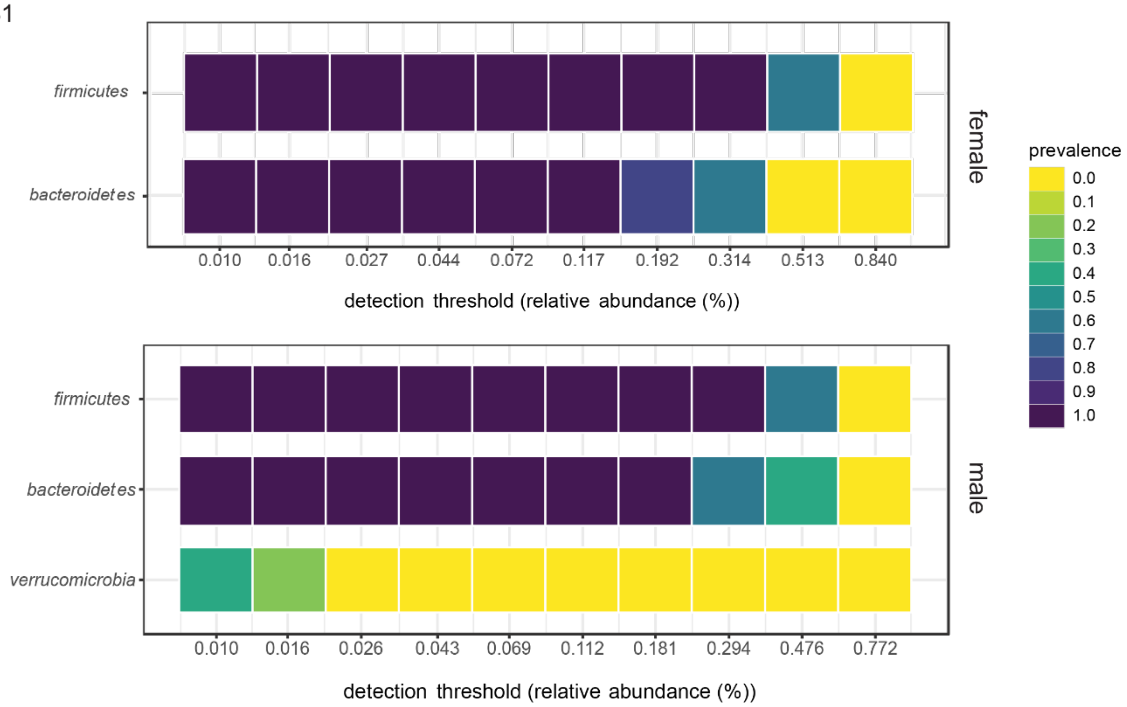
firmicutes
bacteroidetes
female
0.010
0.016
0.027
0.044
0.072
0.117
0.192
0.314
0.513
0.840
detection threshold (relative abundance (%))
firmicutes
bacteroidetes
verrucomicrobia
male
0.010
0.016
0.026
0.043
0.069
0.112
0.181
0.294
0.476
0.772
detection threshold (relative abundance (%))
prevalence
0.0
0.1
0.2
0.3
0.4
0.5
0.6
0.7
0.8
0.9
1.0

S2

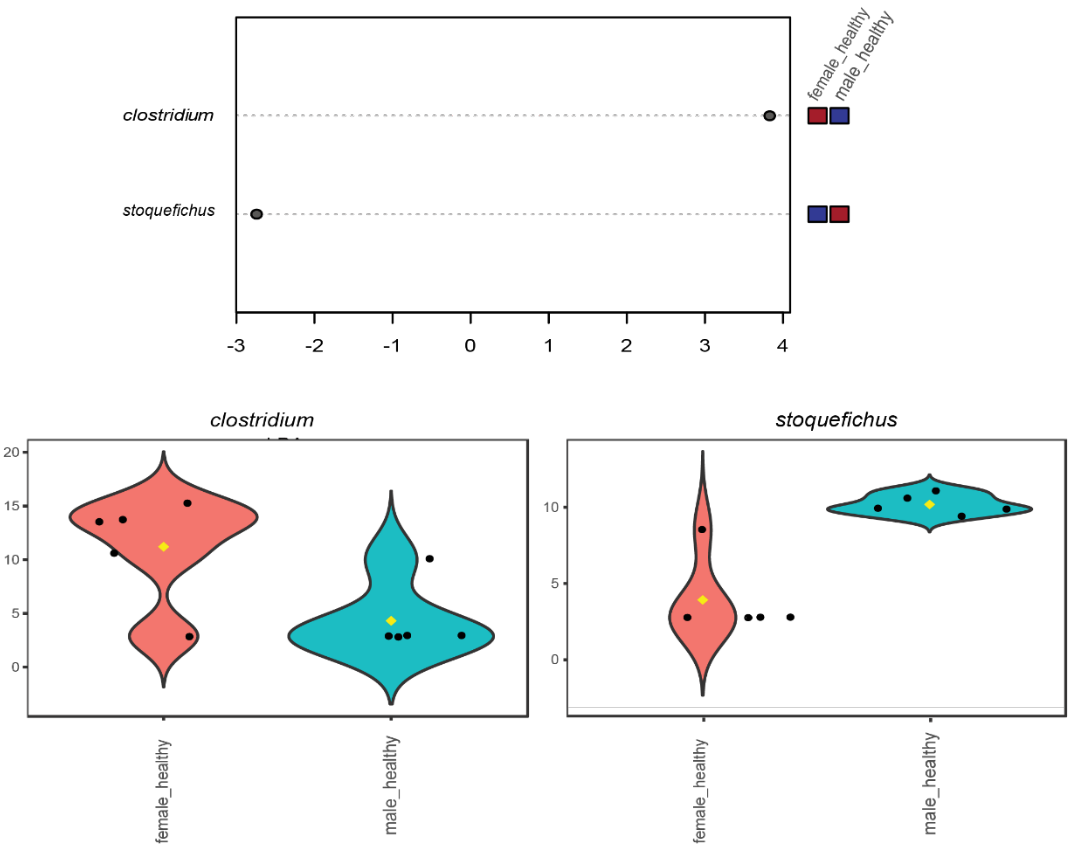
female_healthy
male_healthy
clostridium
stoquefichus
-3
-2
-1
0
1
2
3
4
clostridium
stoquefichus
20
15
10
5
0
female_healthy
male_healthy
10
5
0
female_healthy
male_healthy

Supplementary Figure: S1) Core microbiome abundance analysis of Operational Taxonomic Units (OTUs) at Phylum level. **S2)** Differential taxa at genus level identified by LefSe analysis (LDA >2.0, FDR < 0.05).
